# Supplementary material for: Direct observation of two-channel photodissociation of carbon monoxide from the hemoglobin subunits
Source: Nat Commun. 2025 Aug 20;16:7746. doi: 10.1038/s41467-025-63092-z (PMC12368136; doi:10.1038/s41467-025-63092-z)
Supplement: Supplementary file 1 — Supplementary Information [file 41467_2025_63092_MOESM1_ESM.pdf]

Supplementary Information for

Direct observation of two-channel photodissociation of carbon monoxide from the hemoglobin subunits

Sergei V. Lepeshkevich[1]✉, Igor V. Sazanovich[2]✉, Marina V. Parkhats[1], Syargey N. Gilevich[3], Aliaksei V. Yantsevich[3], Julia A. Weinstein[4], Michael Towrie[2] & Boris M. Dzhagarov[1]

[1]B.I. Stepanov Institute of Physics, National Academy of Sciences of Belarus, 68 Nezavisimosti Ave, Minsk 220072, Belarus.
[2]Central Laser Facility, Research Complex at Harwell, STFC Rutherford Appleton Laboratory, Harwell Campus, OX11 0QX, UK.
[3]Institute of Bioorganic Chemistry, National Academy of Sciences of Belarus, 5/2 Academician V.F. Kuprevich Street, Minsk 220141, Belarus.
[4]University of Sheffield, Department of Chemistry, Dainton Building, Brook Hill, Sheffield S3 7HF, UK.
✉ e-mail: s.lepeshkevich@ifanbel.bas-net.by; igor.sazanovich@stfc.ac.uk

Contents

Supplementary Note 1. Analysis of the transient spectra …………………………… 2
A. Singular value decomposition (SVD) analysis…………………………….. 2
B. Maximum entropy method (MEM) analysis……………………................. 3
C. Spectral changes associated with the fast and slow evolving species……… 3
Supplementary Note 2. Time-dependent absorption anisotropy and rotational diffusion of proteins……………………………………………………….. 5
Supplementary Note 3. Preparation of the isolated hemoglobin chains…..……….. 7
Supplementary Figures………………………………………………………… 8
Supplementary Tables…………………………………………………................... 11
Supplemental References…………………………………………….................. 15

Supplementary Note 1. Analysis of the transient spectra

A. Singular value decomposition (SVD) analysis

The Singular value decomposition analysis was performed in IgorPro software (WaveMetrics, U.S.A.). The transient spectra, $D(\tilde{\nu}, P, t)$, measured at certain wavenumbers $\tilde{\nu}$ at different time delays t for a chosen polarization P can be viewed as the columns of an $m \times n$ matrix **D**, where m is the number of spectral points $\tilde{\nu}$, n is the number of time delays t. In order to describe time courses of spectral changes for each studied heme protein at each polarization, the corresponding data matrix **D** was first subjected to singular value decomposition (SVD)[1] which transforms the matrix **D** into a product of three matrices, $\mathbf{D} = \mathbf{U}\ \mathbf{S}\ \mathbf{V}^{\mathrm{T}}$. Here, **U** is an $m \times n$ matrix of m linearly independent, orthonormal basis spectra U_r ($r = \overline{1,m}$) that describe all the spectra in the data matrix **D**. **S** is an $n \times n$ diagonal matrix with non-negative size-ordered elements called singular values which are a measure of the contribution of the corresponding basis spectrum to the data matrix **D**. $\mathbf{V}^{\mathrm{T}}$ is the transpose of an $n \times n$ matrix **V** having n orthonormal kinetic vectors V_c ($c = \overline{1,n}$), *i.e.* the time evolutions of the corresponding basis spectra. In the present application, only a few basis spectra are expected to make significant contribution to **D**. The remainder can be considered as noise. To determine the SVD components that provide the best least-squared approximation of the data matrix **D**, the magnitudes of their singular values as well as the autocorrelations of the corresponding columns of **U** and **V** were analyzed.[1]

It was found that only the first two SVD components make the main contribution to the transient spectra, $D(\tilde{\nu}, P, t)$, measured for the isolated carbonmonoxy α chains at each polarization setting in the spectral region of 1,880–2,005 cm^{-1} (the region of ground-state bleach). The first two basis spectra, U_1 and U_2, are shown in Supplementary Fig. 2a and 2b, respectively. The time-dependent amplitudes, V_1 and V_2, multiplied by the corresponding singular values, s_1 and s_2, are shown in Supplementary Fig. 2d and 2e, respectively. As it is seen, $s_1\mathrm{V}_1$ makes the dominant contribution being larger by one order of magnitude than the contribution of $s_2\mathrm{V}_2$. The spectral changes, $D(\tilde{\nu}, P, t)$, observed for the isolated carbonmonoxy β chains are more complicated than those for the α chains. For the β chains, the first three SVD components (Supplementary Fig. 3) provide the best representation of the data matrix in the least-square approach. Here, the contribution of $s_1\mathrm{V}_1$ is larger than those of $s_2\mathrm{V}_2$ and $s_3\mathrm{V}_3$ by more than one and two orders of magnitude, respectively. Since all other SVD components contain no real spectral information and correspond to noise with a random time dependence, for further analysis of the data, only the first two SVD components were retained for the α chains and the first three for the β chains. Therefore, the analyzed data matrix **D** may be approximated as $\mathbf{D} \cong \mathbf{D}' = \mathbf{U}'\mathbf{S}'\mathbf{V}'^{\mathrm{T}}$, where $\mathbf{U}'$, $\mathbf{S}'$, and $\mathbf{V}'$ are dimensionally-reduced matrices, which are generated by removing non-signifigant singular components from **U**, **S**, and **V**, respectively. The present SVD analysis provides a model-independent

estimation of the number of distinguishable species in the entire time range. The number of species is equal to the number of principal SVD components. Therefore, for the α and β chains, we identified two and three distinct species, respectively.

B. Maximum entropy method (MEM) analysis

The time-dependent amplitudes, V_i, obtained in the course of the SVD analysis (see Supplementary Note 1A), were subsequently subjected to the maximum entropy method (MEM) analysis[2] which extracts two model-independent distributions of the effective log-lifetimes, $g(\log \tau)$ and $h(\log \tau)$, from the data. For the MEM analysis, the program MemExp (version 3.0)[3,4] was used. The fit $F(t)$ to the amplitudes V_i at time t can be written as

$$F(t) = F_0 \int_{-\infty}^{+\infty} (g(\log \tau) - h(\log \tau)) e^{-t/\tau} d \log \tau \tag{S1}$$

where F_0 is a normalization constant, $g(\log \tau)$ and $h(\log \tau)$ are the lifetime distributions that correspond to decaying and rising kinetics, respectively. The quality of the fit was evaluated by the χ^2 value,[5] the correlation length of the residuals, τ_c,[3] as well as the values of Ratio[6] and TEST.[7] The extracted lifetime distributions for the α and β chains are displayed in the right column of Supplementary Fig. 2 and 3, respectively. For each appreciable peak in every recovered lifetime distribution, the area, a, and the mean, τ, were determined for the α and β chains and listed in Supplementary Table 1 and 2, respectively.

C. Spectral changes associated with the fast and slow evolving species

To simplify the determination of the kinetic model describing the experimental data, spectra associated with certain bands in the lifetime distributions were calculated according to the approach explained in the present Section. It should be stressed that the number of bands and their positions in the lifetime distributions derived by MEM from the amplitudes V_i (see Supplementary Note 1B) depend on both the relative polarization, P, and the type of hemoglobin (Hb) chains (Supplementary Table 1 and 2). However, all the obtained lifetime distributions have one common feature – the band with the smallest characteristic mean lifetime in the range between 8 and 18 ps (referred to in the text as the "fast component"). Hereafter, this band being a part of the lifetime distributions corresponding to decaying and rising kinetics will be designated as $g_{\text{fast}}(\log \tau)$ and $h_{\text{fast}}(\log \tau)$, respectively. The spectral changes associated with this band are defined as:

$$\mathbf{D}_{\text{fast}} = \mathbf{U}'\mathbf{S}'\mathbf{F}_{\text{fast}}^{\mathrm{T}}, \tag{S2}$$

where the columns of $\mathbf{F}_{\text{fast}}$ are a set of kinetic vectors $F_{\text{fast}}(t)$, each of which was calculated for the corresponding basis spectrum U_i as:

$$F_{\text{fast}}(t) = F_0 \int_{\log \tau_{\text{ini}}}^{\log \tau_{\text{fin}}} (g_{\text{fast}}(\log \tau) - h_{\text{fast}}(\log \tau)) e^{-t/\tau} d \log \tau \tag{S3}$$

Here, it is assumed that both $g_{fast}(\log \tau)$ and $h_{fast}(\log \tau)$ are equal to zero for $\log \tau < \log \tau_{ini}$ and $\log \tau > \log \tau_{fin}$ ($\tau_{ini} \sim 10^{-12}$ s, $\tau_{fin} \sim 10^{-10}$ s). The spectral changes associated with the rest of the lifetime distributions (referred to in the text as the "slow components") were determined as:

$$\mathbf{D}_{slow} = \mathbf{D}' - \mathbf{D}_{fast}. \tag{S4}$$

For the isolated α chains, the representative calculated spectral changes associated with the fast component are shown in Fig. 4a (main text), while those associated with the slow components are shown in Fig. 4c (main text). The corresponding spectra calculated for the isolated β chains are presented in Fig. 4b and 4d (main text), respectively. Fig. 4 shows the data obtained at the magic angle polarization setting. Similar spectra were obtained for the other two (parallel and perpendicular) polarization settings.

As it is seen from Fig. 4a,b, the fast component in the lifetime distributions is associated exclusively with positive transient absorption bands. It should be noted that the entirely-positive transient absorption signal (Fig. 4a,b) decays completely by 100 ps and is associated with the intermediate species evolving in the first 100 ps. In turn, the slow components in the lifetime distributions are associated exclusively with negative transient absorption bands (Fig. 4c,d). The entirely-negative transient absorption signal (Fig. 4c,d) decays completely by 800 μs and is associated with the intermediate species evolving over a timescale ranging from nanoseconds to hundreds of microseconds.

Supplementary Note 2. Time-dependent absorption anisotropy and rotational diffusion of proteins

The time-dependent ligand orientation is related to the time-dependent absorption anisotropy, defined as $r(t) = [\Delta A^{\parallel}(t) - \Delta A^{\perp}(t)]/[\Delta A^{\parallel}(t) + 2\Delta A^{\perp}(t)]$, where $\Delta A^{\parallel}$, and $\Delta A^{\perp}$ are the pump-induced changes in the absorbance spectra measured parallel and perpendicular to the photolysis polarization direction, respectively.

To determine the time-dependent anisotropy $r(t)$ of the ground-state bleach spectra, the following procedure was performed. The time-dependent amplitudes s_1V_1 (Fig. 5b or 6b, main text), obtained by global SVD of the spectra associated with the slow evolving species at the three polarization settings, were globally fitted to:

$$\begin{bmatrix} F_{\mathrm{ma}}(t) \\ F_{\|}(t) \\ F_{\perp}(t) \end{bmatrix} = \begin{bmatrix} 1 \\ 1+2r(t) \\ 1-r(t) \end{bmatrix} \cdot F_0 \int_{-\infty}^{+\infty} g_{\mathrm{ma}}(\log \tau) e^{-t/\tau} d \log \tau , \quad \text{(S5)}$$

where $F_{\mathrm{ma}}(t)$, $F_{\|}(t)$, and $F_{\perp}(t)$ are the fit to the vector s_1V_1 for the magic angle, parallel, and perpendicular polarization setting, respectively; F_0 is the normalization constant; $g_{\mathrm{ma}}(\log \tau)$ is the lifetime distribution derived by MEM from the vector s_1V_1 for the magic angle polarization setting. For the anisotropy time dependence, $r(t)$, an exponential decay model was applied:

$$r(t) = r_0 \cdot \exp(-t/\tau_{\mathrm{rot}}) \quad \text{(S6)}$$

where r_0 is the anisotropy at time zero (the initial anisotropy) before any protein or heme motion occurs, τ_{rot} is the rotational correlation time. The fitted parameters r_0 and τ_{rot} are listed in Supplementary Table 5. For both the isolated α and β chains of human Hb, the initial anisotropy, r_0, was found to be equal, within the experimental accuracy, to –0.174, which agrees well with the data determined previously for tetrameric Hb.[8] The rotational correlation times, τ_{rot}, obtained in the present work are in agreement with those reported earlier.[9,10,11]

The time-dependent anisotropy $r(t)$ of the excited-state absorption spectra consisting of the E_0 and E_1 vibrational bands was also obtained in a similar fashion as that described above. In particular, the time-dependent amplitudes s_1V_1 (Fig. 5a or 6a, main text), obtained by global SVD of the spectra associated with the fast evolving species at the three polarization settings, were also globally fitted to eqn (S5), where $r(t)$ was described by eqn (S6). However, in the case of the anisotropy of the excited-state absorption spectra, τ_{rot} in eqn (S6) was fixed at the value found above for the anisotropy of the ground-state bleach spectra. For both Hb chains, the initial anisotropy, r_0, of the excited-state absorption spectra was found to be equal to –0.166 (Supplementary Table 5).

The initial anisotropy r_0 of the vibrational spectra for photolyzed CO located in the primary docking site was determined directly using the pump-induced changes in the absorbance spectra (in the

vicinity of the peaks of the B_1 and B_2 bands) measured parallel ($\Delta A^{\parallel}$) and perpendicular ($\Delta A^{\perp}$) to the photolysis polarization direction on the picosecond timescale. For both Hb chains, the initial anisotropy, r_0, of the vibrational spectra for photolyzed CO in both B_1 and B_2 states was found to be equal to 0.06 ± 0.02.

The initial anisotropy r_0 were used to obtain the geometry of both the bound and photolyzed CO in the Hb chains. The absorption Q-band of the carbonmonoxy-heme proteins (absorption maximum at 540 nm) was chosen for the photolysis because this absorption is isotropic in the heme plane.[12] Under the chosen excitation conditions, the polarization anisotropy r_0 is sensitive to the angle between the CO molecule and the heme normal. Assuming that the IR transition dipole of the CO molecule is along the CO axis, the average angle Θ between the CO bond and the heme plane normal were determined from the value of r_0 by the following equation[13,14]:

$$\Theta = \arcsin\sqrt{10\left(r_0 + 0.2\right)/3}\,. \qquad \text{(S7)}$$

Supplementary Note 3. Preparation of the isolated hemoglobin chains

Hb was purified from freshly drawn blood. The isolated α and β chains were subsequently obtained in the carbonmonoxy form by the *p*-mercuribenzoate (PMB) method[15] with minor modifications. Separation of the isolated chains with bound PMB (α^{PMB} and β^{PMB} chains) was performed by ion-exchange chromatography on a column with DEAE-Sepharose CL-6B (GE Healthcare) equilibrated with 20 mM Tris HCl buffer, pH 8.1. Elution was carried out by a linear gradient from 20 mM to 380 mM Tris HCl buffers, both at pH 8.1, in a gradient mixer GM-1 (GE Healthcare). Under the specified conditions, the α^{PMB} chains were eluted earlier than the β^{PMB} chains. The isolated α^{PMB} and β^{PMB} chains were regenerated to their -SH forms (α^{SH} and β^{SH} chains) using dithiothreitol (DTT).[16] PMB was removed from the α chains after incubation with DTT for a few minutes and subsequent passage through a Sephadex G-25 column equilibrated with 5 mM Tris HCl buffer, pH 8.1. The β^{PMB} solution was incubated with DTT for 30 minutes on ice and, subsequently, applied to a DEAE-Sepharose CL-6B column equilibrated with 10 mM Tris HCl buffer, pH 8.1. The β^{PMB} chains were retained at the top of the column. The column was washed with the same buffer containing 4 mM DTT for ~2.5 hrs and left overnight. The next morning, the column was washed with 140 mM Tris HCl buffer, pH 8.1, for ~2 hrs, then the β^{SH} chains were eluted with 220 mM Tris HCl buffer at the same pH. All the buffers used for the sulfhydryl groups regeneration were purged carefully with nitrogen. Finally, the isolated α^{SH} and β^{SH} chains were concentrated and dialyzed against 50 mM Tris buffer, pD 8.2. All the procedures were carried out at 4°C. The identity of the α and β chains were verified by electrospray ionization mass spectroscopy.

Protein samples for experiments were prepared using 50 mM deuterated Tris buffer, pD 8.2, at 19° C under 1 bar atmosphere of carbon monoxide (CO). CO gas was supplied by CK Special Gases Limited UK (99.97% purity). D_2O was used to avoid strong water absorption in the spectral region of interest. Concentrations of the isolated carbonmonoxy α^{SH} and β^{SH} chains were 3.0 and 4.0 mM in heme, respectively. The protein samples were controlled before and after the experiment by UV-Vis and FT-IR spectroscopy.

Supplementary Figures

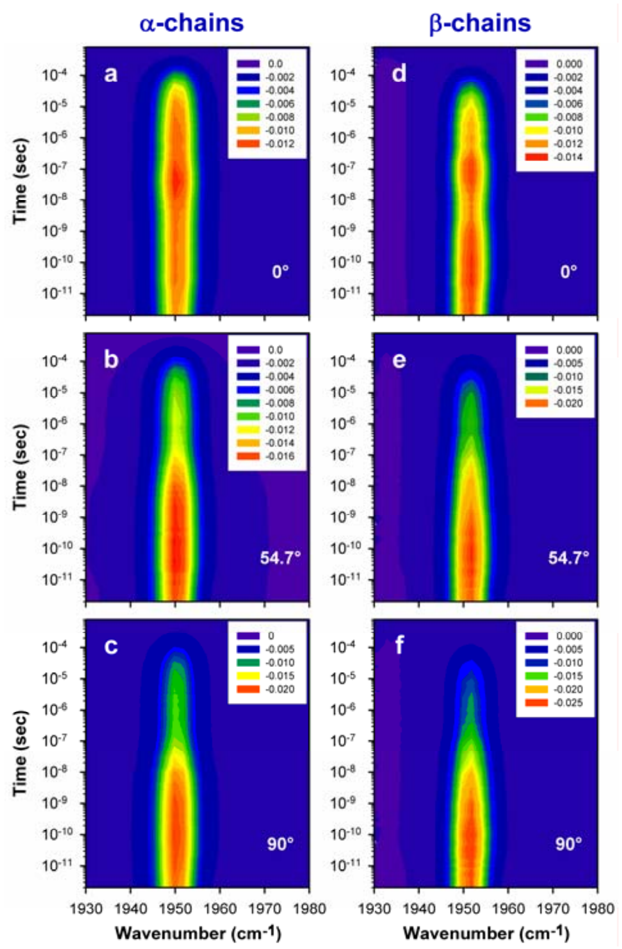

Supplementary Figure 1 | Time-resolved polarized transient IR absorption spectra. a–f Contour plots of the spectra after the photoexcitation of the isolated carbonmonoxy α chains (**a**–**c**) and carbonmonoxy β chains (**d**–**f**). The relative polarization of the pump and probe beams: 0° (**a**, **d**), 54.7° (**b**, **e**), and 90° (**c**, **f**). Conditions: 50 mM Tris buffer, pD 8.2, at 19°C. Concentrations of the carbonmonoxy α and β chains were 3.0 and 4.0 mM in heme, respectively. Excitation wavelength, λ_{exc} = 543 nm. Source data are provided as a Source Data file.

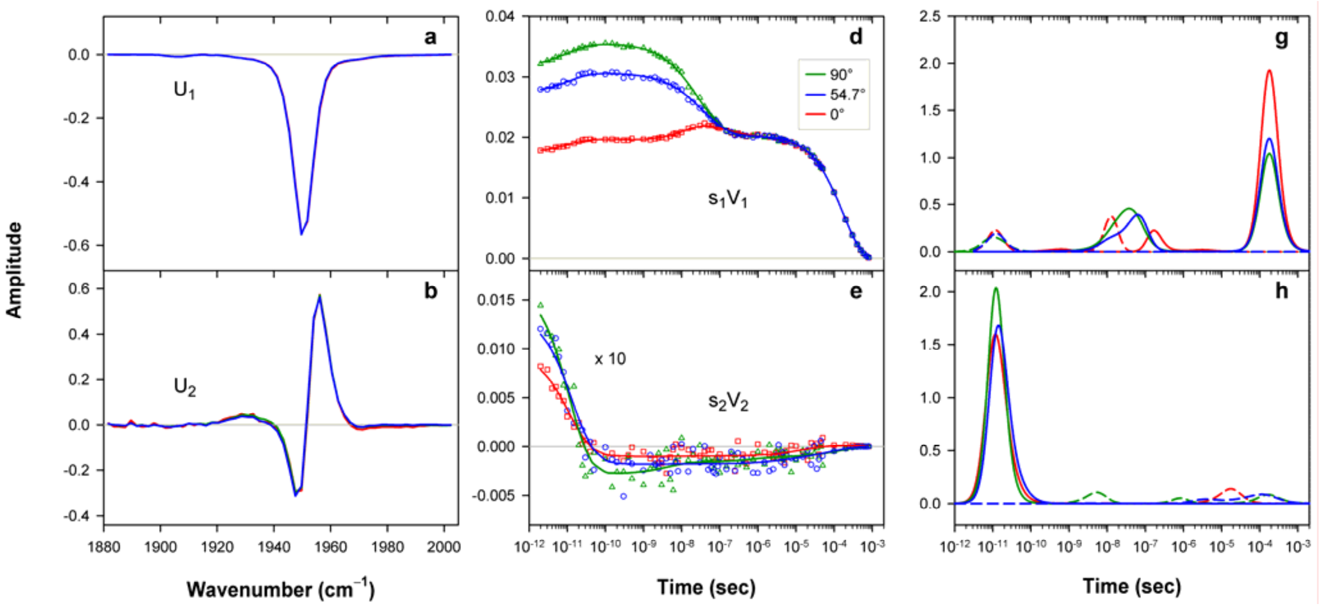

Supplementary Figure 2 | SVD of the measured time-resolved polarized transient IR absorption spectra *D*($\tilde{\nu}$, *P*, *t*) after the photoexcitation of the isolated carbonmonoxy α chains. The first two SVD components that make the main contribution to the observed spectra are shown. **a**, **b** Orthonormal basis spectra (columns of **U**). **d**, **e** Time-dependent amplitudes (columns of **V**) multiplied by the corresponding singular values (elements of the diagonal matrix **S**). The time-dependent amplitudes and their fit obtained with the MEM analysis[2] (see Supplementary Note 1B for details) are reported as symbols and solid lines, respectively. **g** and **h** Lifetime distributions derived by MEM from V_1 and V_2, respectively. The lifetime distributions, corresponding to decaying and rising kinetics, are presented as solid and dash lines, respectively. The data obtained at the three polarization settings are colour-coded: 0° (red), 54.7° (blue), and 90° (green). Source data are provided as a Source Data file.

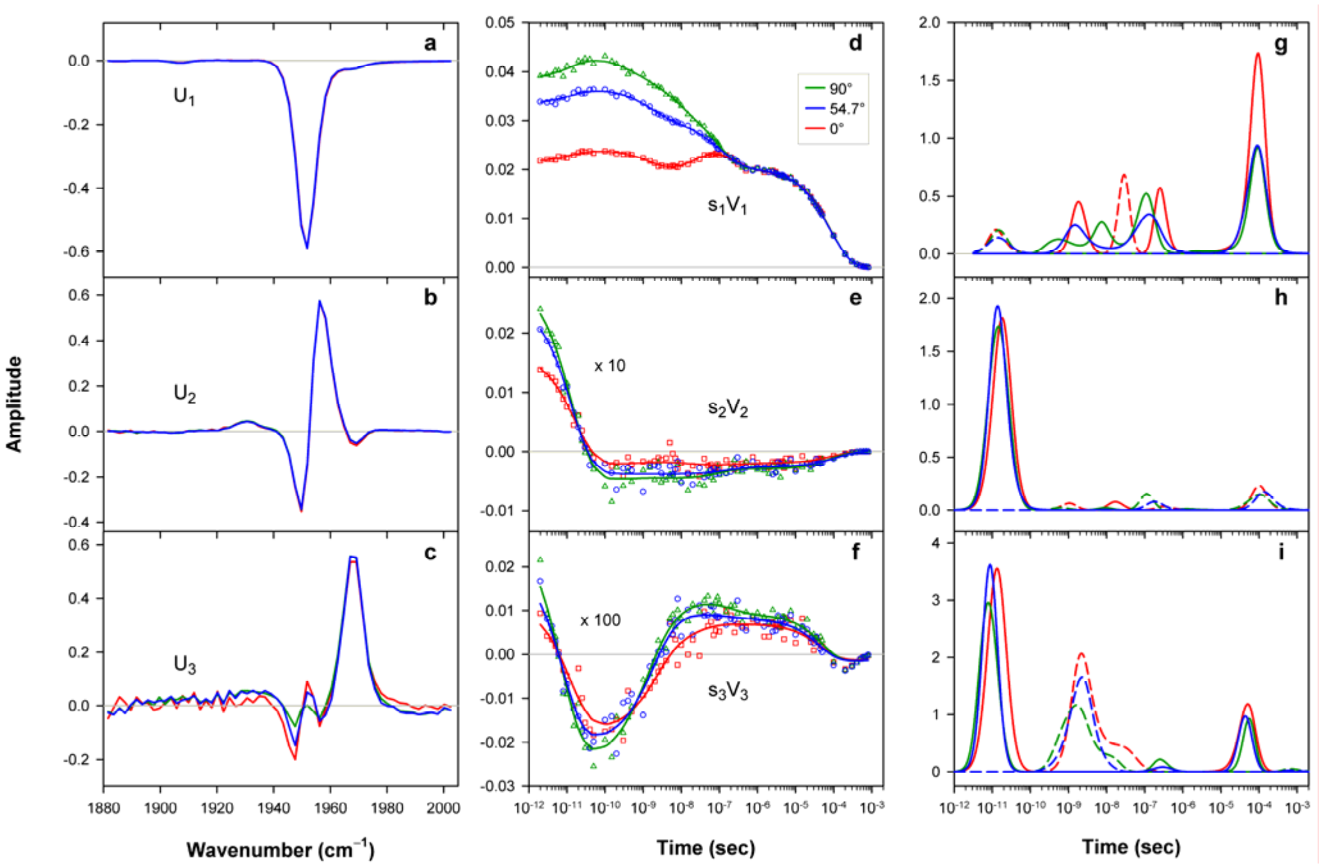

Supplementary Figure 3 | SVD of the measured time-resolved polarized transient IR absorption spectra $D(\tilde{\nu}, P, t)$ after the photoexcitation of the isolated carbonmonoxy β chains. The first three SVD components that make the main contribution to the observed spectra are shown. **a**–**c** Orthonormal basis spectra (columns of **U**). **d**–**f** Time-dependent amplitudes (columns of **V**) multiplied by the corresponding singular values (elements of the diagonal matrix **S**). **g**, **h**, and **i** Lifetime distributions derived by MEM from V_1, V_2, and V_3, respectively. Description of symbols used is the same as for Supplementary Fig. 2. Source data are provided as a Source Data file.

Supplementary Tables

Supplementary Table 1 | MEM-derived parameters characterizing the lifetime distributions obtained for the time-dependent amplitudes, V_i, of the basis spectra for the isolated carbonmonoxy α chains

	P	**τ_1 (rise, ps)**	**τ_2 (rise, ns)**	**τ_3 (decay, ns)**	**τ_4 (decay, μs)**	***a_1***	***a_2***	***a_3***	***a_4***
**V_1**	0°	12	13	188	185	–0.120	–0.172	0.114	1.14
	54.7°	14	–	35	183	–0.120	–	0.37	0.75
	90°	12	–	30	185	–0.123	–	0.47	0.65

	P	**τ_1 (decay, ps)**	**τ_2 (rise, ns)**	**τ_3 (rise, μs)**	***a_1***	***a_2***	***a_3***
**V_2**	0°	13	–	14	1.13	–	–0.114
	54.7°	16	–	97	1.10	–	–0.098
	90°	13	4.6	160	1.21	–0.077	–0.073

Here, *P* is the relative polarization of the pump and probe beams; τ_i and a_i are the mean and the area of the *i*th MEM peak, respectively. The mean, τ_i, and the area, a_i, were determined according to eqn (8) and (7) from ref. 3, respectively. Positive and negative area values correspond to decaying and rising kinetics, respectively.

Supplementary Table 2 | MEM-derived parameters characterizing the lifetime distributions obtained for the time-dependent amplitudes, V_i, of the basis spectra for the isolated carbonmonoxy β chains

	P	τ_1 (rise, ps)	τ_2 (decay, ns)	τ_3 (ns)	τ_4 (decay, μs)	τ_5 (decay, μs)	a_1	a_2	a_3	a_4	a_5
	0°	14	1.8	29	0.27	85	−0.117	0.21	−0.26	0.22	0.93
**V_1**	54.7°	17	1.8	–	0.115	85	−0.097	0.193	–	0.30	0.61
	90°	16	0.57	7.2	0.101	86	−0.116	0.100	0.170	0.31	0.52

	P	τ_1 (decay, ps)	τ_2 (rise, ns)	τ_3 (decay, ns)	τ_4 (rise, μs)	τ_5 (rise, μs)	a_1	a_2	a_3	a_4	a_5
	0°	18	0.97	17	0.39	104	1.18	−0.036	0.044	−0.022	−0.130
**V_2**	54.7°	15	–	–	0.18	149	1.23	–	–	−0.055	−0.119
	90°	14	–	–	0.114	100	1.22	–	–	−0.072	−0.113

	P	τ_1 (decay, ps)	τ_2 (rise, ns)	τ_3 (decay, μs)	τ_4 (decay, μs)	a_1	a_2	a_3	a_4
	0°	13	4.0	–	46	2.5	−2.1	–	0.73
**V_3**	54.7°	9	2.1	0.29	43	2.3	−1.63	0.063	0.55
	90°	8	1.9	0.27	53	1.89	−1.31	0.115	0.40

Here, *P* is the relative polarization of the pump and probe beams; τ_i and a_i are the mean and the area of the *i*th MEM peak, respectively. The mean, τ_i, and the area, a_i, were determined according to eqn (8) and (7) from ref. 3, respectively. Positive and negative area values correspond to decaying and rising kinetics, respectively.

Supplementary Table 3 | Spectroscopic parameters of the A_0 and A_1 conformational substates obtained by fitting the ground-state FTIR absorption spectra as well as the first basis spectra, U_1 (derived from the global SVD of the transient IR absorption spectra associated with the slow evolving species at three polarization settings), for the isolated carbonmonoxy hemoglobin chains in terms of two voigtian components

**Protein**	**A_1**			**A_0**		
	**Area (%)**	**ν_{CO} (cm^{-1})**	**$\Delta\nu$ (cm^{-1})**	**Area (%)**	**ν_{CO} (cm^{-1})**	**$\Delta\nu$ (cm^{-1})**
αCO*	100	1,950.0 ± 0.2	7.6 ± 0.5			
αCO**	100	1,950.3 ± 0.2	8.4 ± 0.7			
βCO*	96 ± 1	1,951.5 ± 0.2	7.4 ± 0.3	4 ± 1	1,968.8 ± 1.0	12 ± 2
βCO**	95 ± 2	1,951.5 ± 0.2	8 ± 1	5 ± 2	1,966.1 ± 1.4	12 ± 4

Here, Area is the relative area of the band; ν_{CO} is the position of the peak maximum; $\Delta\nu$ is the full width at half maximum. *The parameters were derived from the ground-state FTIR spectra. **The parameters were derived from the first basis spectra, U_1. The uncertainties are presented as 95% confidence intervals.

Supplementary Table 4 | The MEM-derived parameters for CO rebinding to the isolated α and β chains in the A_1 conformational substate

**Protein**	**τ_1 (ns)**	**τ_2 (ns)**	**τ_3 (ms)**	**F_1 (× 10^{-2})**	**F_2 (× 10^{-2})**	**F_3 (× 10^{-2})**
αCO	34	–	0.18	33	–	67
βCO	1.7	115	0.085	18	27	55

Here, τ_i $(i=\overline{1,3})$ is the mean of the ith MEM peak; F_i $(i=\overline{1,3})$ is the fractional contribution of the ith MEM peak to the total peaks area. All the fractional contributions are normalized to unity. In this case, F_1 and F_2 determine the fraction for the fast and slow geminate phase, respectively. The sum of F_1 and F_2 is equal to the fraction of geminate CO rebinding. In turn, F_3 determines the fraction of bimolecular CO rebinding, which is equal to the efficiency of CO escape from the protein matrix after photodissociation.

Supplementary Table 5 | Structural and kinetic parameters for the A_1 conformational substate and the E_0 and E_1 excited states

Protein	τ_{rot} (ns)	A_1		E_0, E_1			
		r_0	Θ (degrees)	r_0	Θ (degrees)	τ^a (ps)	τ^b (ps)
αCO	26 ± 1	−0.174 ± 0.002	17 ± 1	−0.166 ± 0.007	20 ± 2	13	14.2 ± 1.1
βCO	48 ± 3	−0.173 ± 0.002	17 ± 1	−0.166 ± 0.013	20 ± 4	15	16.5 ± 1.7

Here, τ_{rot} is the rotational correlation time; r_0 is the initial anisotropy; Θ is the angle between the CO axis and the normal to the heme plane; τ is the excited state lifetime.

[a] The excited state lifetime obtained by MEM analysis.

[b] The excited state lifetime obtained by a single-exponential fitting.

The uncertainties are presented as 95% confidence intervals.

Supplementary Table 6 | Spectroscopic parameters of the E_0 and E_1 excited states obtained by fitting the first basis spectra, U_1 (derived from the global SVD of the transient IR absorption spectra associated with the fast evolving species at three polarization settings), for the isolated carbonmonoxy hemoglobin chains in terms of two voigtian components

Protein	E_0			E_1			$\nu_{CO}(E_0) - \nu_{CO}(E_1)$ (cm^{-1})
	Area (%)	ν_{CO} (cm^{-1})	Δν (cm^{-1})	Area (%)	ν_{CO} (cm^{-1})	Δν (cm^{-1})	
αCO	94 ± 2	1,953.2 ± 0.2	10 ± 1	6 ± 2	1,931.5 ± 1.3	13 ± 4	21.7 ± 1.3
βCO	96 ± 2	1,955.2 ± 0.2	9 ± 1	4 ± 2	1,929.8 ± 1.2	8 ± 3	25.4 ± 1.2

Here, Area is the relative area of the band; ν_{CO} is the position of the peak maximum; Δν is the full width at half maximum. The uncertainties are presented as 95% confidence intervals.

Supplemental References

1. Henry, E. R. & Hofrichter, J. Singular value decomposition: application to analysis of experimental data. *Methods Enzymol*. **210**, 129–192 (1992).
2. Steinbach, P. J. et al. Determination of rate distributions from kinetic experiments. *Biophys. J.* **61**, 235–245 (1992).
3. Steinbach, P. S., Ionescu, R. & Matthews, C. R. Analysis of kinetics using a hybrid maximum-entropy/nonlinear-least-squares methods: application to protein folding. *Biophys. J.* **82**, 2244–2255 (2002).
4. Steinbach, P. J. Inferring lifetime distributions from kinetics by maximizing entropy using a bootstrapped model. *J. Chem. Inf. Comput. Sci*. **42**, 1476−1478 (2002).
5. Lavalette, D., Tetreau, C., Brochon, J.-C. & Livesey, A. Conformational fluctuations and protein reactivity: determination of the rate-constant spectrum and consequences in elementary biochemical processes. *Eur. J. Biochem*. **196**, 591–598 (1991).
6. Cornwell, T. J. & Evans, K. F. A simple maximum entropy deconvolution algorithm. *Astron. Astrophys*. **143**, 77–83 (1985).
7. Skilling, J. & Bryan, R. K. Maximum entropy image reconstruction: general algorithm. *Mon. Not. R. Astr. Soc*. **211**, 111–124 (1984).
8. Locke, B., Lian, T. & Hochstrasser, R. M. Determination of Fe−CO geometry and heme rigidity in carbonmonoxyhemoglobin using femtosecond IR spectroscopy. *Chem. Phys*. **158**, 409–419 (1991).
9. Jones, C. M. et al. Speed of intersubunit communication in proteins. *Biochemistry* **31**, 6692−6702 (1992).
10. Gilman, J. G. Carbon-13 nuclear magnetic resonance study of the motional behavior of ethyl isocyanide bound to myoglobin and hemoglobin. *Biochemistry* **18**, 2273−2279 (1979).
11. Madrid, M., Simplaceanu, V., Ho, N. T. & Ho, C. Effects of chemical exchange and dipole-dipole interactions on the proton relaxation rates of surface histidyl residues in human hemoglobin. *J. Magn. Reson*. **88**, 42−59 (1990).
12. Eaton, W. A., Hanson, L. K., Stephens, P. J., Sutherland, J. C. & Dunn, J. B. R. Optical spectra of oxy- and deoxyhemoglobin. *J. Am. Chem. Soc*. **100**, 4991–5003 (1978).
13. Moore, J. N., Hansen, P. A. & Hochstrasser, R. M. Iron-carbonyl bond geometries of carboxymyoglobin and carboxyhemoglobin in solution determined by picosecond time-resolved infrared spectroscopy. *Proc. Natl Acad. Sci. USA* **85**, 5062–5066 (1988).
14. Ormos, P. et al. Orientation of carbon monoxide and structure-function relationship in carbonmonoxymyoglobin. *Proc. Natl Acad. Sci. USA* **85**, 8492–8496 (1988).

15. Bucci, E. & Fronticelli, C. A new method for the preparation of α and β subunits of human hemoglobin. *J. Biol. Chem.* **240**, 551−552 (1965).
16. Parkhurst, K. M. & Parkhurst, L. J. Rapid preparation of native alpha and beta chains of human hemoglobin. *Int. J. Biochem.* **24**, 993−998 (1992).
