# Supplementary material for: Direct observation of two-channel photodissociation of carbon monoxide from the hemoglobin subunits
Source: Nat Commun. 2025 Aug 20;16:7746. doi: 10.1038/s41467-025-63092-z (PMC12368136; doi:10.1038/s41467-025-63092-z)
Supplement: Supplementary file 2 — Reporting Summary [file 41467_2025_63092_MOESM2_ESM.pdf]

nature portfolio

Corresponding author(s): Dr. Sergei V. Lepeshkevich; Dr. Igor V. Sazanovich

Last updated by author(s): 02 July 2025

Reporting Summary

Nature Portfolio wishes to improve the reproducibility of the work that we publish. This form provides structure for consistency and transparency in reporting. For further information on Nature Portfolio policies, see our Editorial Policies and the Editorial Policy Checklist.

Statistics

For all statistical analyses, confirm that the following items are present in the figure legend, table legend, main text, or Methods section.

n/a	Confirmed	
☐	☒	The exact sample size (*n*) for each experimental group/condition, given as a discrete number and unit of measurement
☒	☐	A statement on whether measurements were taken from distinct samples or whether the same sample was measured repeatedly
☒	☐	The statistical test(s) used AND whether they are one- or two-sided<br>*Only common tests should be described solely by name; describe more complex techniques in the Methods section.*
☒	☐	A description of all covariates tested
☒	☐	A description of any assumptions or corrections, such as tests of normality and adjustment for multiple comparisons
☐	☒	A full description of the statistical parameters including central tendency (e.g. means) or other basic estimates (e.g. regression coefficient) AND variation (e.g. standard deviation) or associated estimates of uncertainty (e.g. confidence intervals)
☒	☐	For null hypothesis testing, the test statistic (e.g. *F*, *t*, *r*) with confidence intervals, effect sizes, degrees of freedom and *P* value noted<br>*Give P values as exact values whenever suitable.*
☒	☐	For Bayesian analysis, information on the choice of priors and Markov chain Monte Carlo settings
☒	☐	For hierarchical and complex designs, identification of the appropriate level for tests and full reporting of outcomes
☒	☐	Estimates of effect sizes (e.g. Cohen's *d*, Pearson's *r*), indicating how they were calculated

Our web collection on statistics for biologists contains articles on many of the points above.

Software and code

Policy information about availability of computer code

Data collection	Only the custom software controlling the experimental apparatus was used in the course of data acquisition.
Data analysis	For the data analysis the commercially-available software IgorPro and freely-available software MemExp (version 3.0) were used.

For manuscripts utilizing custom algorithms or software that are central to the research but not yet described in published literature, software must be made available to editors and reviewers. We strongly encourage code deposition in a community repository (e.g. GitHub). See the Nature Portfolio guidelines for submitting code & software for further information.

Data

Policy information about availability of data

All manuscripts must include a data availability statement. This statement should provide the following information, where applicable:

- Accession codes, unique identifiers, or web links for publicly available datasets
- A description of any restrictions on data availability
- For clinical datasets or third party data, please ensure that the statement adheres to our policy

The Source Data file "Lepeshkevich_et_al_Source Data_final_submission_NCOMMS-24-67838.xls" has been supplied which contains all data presented in graphs within the Figures in the main manuscript and in the Supplementary Information. The time-resolved infrared data our paper is based upon has been uploaded into eData data repository and is freely available at https://doi.org/10.5286/edata/947

Research involving human participants, their data, or biological material

Policy information about studies with human participants or human data. See also policy information about sex, gender (identity/presentation), and sexual orientation and race, ethnicity and racism.

Reporting on sex and gender	This information has not been collected.
Reporting on race, ethnicity, or other socially relevant groupings	This information has not been collected.
Population characteristics	No human research participants have been involved in this study.
Recruitment	No human research participants have been involved in this study.
Ethics oversight	Ethics approval was not required for this study.

Note that full information on the approval of the study protocol must also be provided in the manuscript.

Field-specific reporting

Please select the one below that is the best fit for your research. If you are not sure, read the appropriate sections before making your selection.

[x] Life sciences [ ] Behavioural & social sciences [ ] Ecological, evolutionary & environmental sciences

For a reference copy of the document with all sections, see nature.com/documents/nr-reporting-summary-flat.pdf

Life sciences study design

All studies must disclose on these points even when the disclosure is negative.

Sample size	The sample size in time-resolved infrared experiments is determined by the number of time delay points in time domain, and by the number of averaged probing laser shots which defines th ultimate signal to noise ratio for each time-resolved spectrum at a given delay. With respect to the number of used time delay points, we used typically 75 time delay points for the data in the spectral range of Fe-CO stretch vibration. As for the number of acquired laser shots, each time-resolved infrared spectrum at a given time delay is acquired with 150,000 laser shots.
Data exclusions	No data were excluded from the analyses
Replication	The time-resolved infrared measurements were repeated 4 times for each sample, with good agreement between the repeats. The ground-state UV-Visible absorption spectra for alpha chains were repeated 5 times in D2O and 2 times in H2O with good agreement between all repeats. The ground-state UV-Visible absorption spectra for beta chains were repeated 6 times in D2O and 2 times in H2O, with good agreement between all repeats.
Randomization	The time delay points used in the process of time-resolved infrared data acquisition, were scanned in the random order to avoid any systematic effect of potential sample photodegradation on the acquired data.
Blinding	Blinding was not relevant to this study.

Reporting for specific materials, systems and methods

We require information from authors about some types of materials, experimental systems and methods used in many studies. Here, indicate whether each material, system or method listed is relevant to your study. If you are not sure if a list item applies to your research, read the appropriate section before selecting a response.

Materials & experimental systems

n/a	Involved in the study	
[x]	[ ]	Antibodies
[x]	[ ]	Eukaryotic cell lines
[x]	[ ]	Palaeontology and archaeology
[x]	[ ]	Animals and other organisms
[x]	[ ]	Clinical data
[x]	[ ]	Dual use research of concern
[x]	[ ]	Plants

Methods

n/a	Involved in the study	
[x]	[ ]	ChIP-seq
[x]	[ ]	Flow cytometry
[x]	[ ]	MRI-based neuroimaging

Plants

Seed stocks	*Report on the source of all seed stocks or other plant material used. If applicable, state the seed stock centre and catalogue number. If plant specimens were collected from the field, describe the collection location, date and sampling procedures.*
Novel plant genotypes	*Describe the methods by which all novel plant genotypes were produced. This includes those generated by transgenic approaches, gene editing, chemical/radiation-based mutagenesis and hybridization. For transgenic lines, describe the transformation method, the number of independent lines analyzed and the generation upon which experiments were performed. For gene-edited lines, describe the editor used, the endogenous sequence targeted for editing, the targeting guide RNA sequence (if applicable) and how the editor was applied.*
Authentication	*Describe any authentication procedures for each seed stock used or novel genotype generated. Describe any experiments used to assess the effect of a mutation and, where applicable, how potential secondary effects (e.g. second site T-DNA insertions, mosiacism, off-target gene editing) were examined.*
